# Late-onset Pompe disease (LOPD) in Belgium: clinical characteristics and outcome measures

**DOI:** 10.1186/s13023-020-01353-4

**Published:** 2020-04-05

**Authors:** P. Vanherpe, S. Fieuws, A. D’Hondt, C. Bleyenheuft, P. Demaerel, J. De Bleecker, P. Van den Bergh, J. Baets, G. Remiche, K. Verhoeven, S. Delstanche, M. Toussaint, B. Buyse, P. Van Damme, C. E. Depuydt, K. G. Claeys

**Affiliations:** 1 Department of Neurology, Neuromuscular Reference Centre, University Hospitals Leuven, Herestraat 49, 3000 Leuven, Belgium; 2 KU Leuven – University of Leuven, Interuniversity Institute for Biostatistics and Statistical Bioinformatics, Leuven, Belgium; 3 Sciensano, Brussels, Belgium; 4 Department of Radiology, University Hospitals Leuven, Leuven, Belgium; 5 Department of Neurology, Neuromuscular Reference Centre, University Hospital Gent, Gent, Belgium; 6 Department of Neurology, Neuromuscular Reference Centre, University Hospital Saint-Luc, Brussels, Belgium; 7 Department of Neurology, Neuromuscular Reference Centre, University Hospital Antwerpen, Antwerpen, Belgium; 8 Department of Neurology, Neuromuscular Reference Centre, University Hospital Erasme, Université Libre de Bruxelles, Brussels, Belgium; 9 Department of Neurology, AZ Sint-Jan Brugge, Brugge, Belgium; 10 Department of Neurology, Neuromuscular Reference Centre of Liège, CHU Liège, Liège, Belgium; 11 Department of Rehabilitation, Centre for Home Mechanical Ventilation and Neuromuscular Reference Centre, Rehabilitation Hospital Inkendaal, Brussels, Belgium; 12 Department of Pulmonology, Leuven University Centre for Sleep and Wake Disorders, University Hospitals Leuven, Leuven, Belgium; 13 VIB, Center for Brain & Disease Research, Laboratory of Neurobiology, Leuven, Belgium; 14 Department of Neurosciences – Experimental Neurology, Laboratory for Muscle Diseases and Neuropathies, KU Leuven, Leuven, Belgium

**Keywords:** Glycogen storage disease type 2, GSD2, Belgian cohort, ActivLim, 6MWD, Respiratory

## Abstract

**Background:**

Late-onset Pompe disease (LOPD) is a rare, hereditary, progressive disorder that is usually characterized by limb-girdle muscle weakness and/or respiratory insufficiency. LOPD is caused by mutations in the acid alpha-glucosidase (*GAA*) gene and treated with enzyme replacement therapy (ERT).

**Methods:**

We studied the clinical, brain imaging, and genetic features of the Belgian cohort of late-onset Pompe disease patients (*N* = 52), and explored the sensitivity of different outcome measures, during a longitudinal period of 7 years (2010–2017), including the activity limitations ActivLim score, 6 min walking distance (6MWD), 10 m walk test (10MWT), MRC sum score, and forced vital capacity (FVC) sitting/supine.

**Results:**

In Belgium, we calculated an LOPD prevalence of 3.9 per million. Mean age at onset of 52 LOPD patients was 28.9 years (SD: 15.8 y), ranging from 7 months to 68 years. Seventy-five percent (*N* = 39) of the patients initially presented with limb-girdle weakness, whereas in 13% (*N* = 7) respiratory symptoms were the only initial symptom. Non-invasive ventilation (NIV) was started in 37% (*N* = 19), at a mean age of 49.5 years (SD: 11.9 y), with a mean duration of 15 years (SD: 10.2 y) after symptom onset. Brain imaging revealed abnormalities in 25% (*N* = 8) of the patients, with the presence of small cerebral aneurysm(s) in two patients and a vertebrobasilar dolichoectasia in another two. Mean diagnostic delay was 12.9 years. All patients were compound heterozygotes with the most prevalent mutation being c.-32-13 T > G in 96%. We identified two novel mutations in *GAA*: c.1610_1611delA and c.186dup11. For the 6MWD, MRC sum score, FVC sitting and FVC supine, we measured a significant decrease over time (*p* = 0.0002, *p* = 0.0001, *p* = 0.0077, *p* = 0.0151), which was not revealed with the ActivLim score and 10MWT (*p* > 0.05).

**Conclusions:**

Awareness on LOPD should even be further increased because of the long diagnostic delay. The 6MWD, but not the ActivLim score, is a sensitive outcome measure to follow up LOPD patients.

## Introduction

Pompe disease, also known as glycogen storage disease type 2 (GSD2) or acid maltase deficiency, is an autosomal recessive disorder caused by a deficiency of the lysosomal enzyme acid alpha-glucosidase (GAA), resulting in the accumulation of glycogen in muscle cells. This usually leads to progressive limb-girdle muscle weakness and respiratory insufficiency, but heart, liver and the nervous system can also be affected [1–5]. Depending on the residual GAA enzyme activity, the disease either develops during the first months of life as the classic infantile Pompe disease (IOPD) [6], or later in life (childhood, adolescence or adulthood) with a milder phenotype known as late-onset Pompe disease (LOPD) [3]. Glycogen storage disease type 2 is a rare disorder, with an estimated prevalence of 1:283,000 in Europe [7]. Over 200 different mutations in the *GAA* gene have been described so far [8]. Current treatment consists of enzyme replacement therapy (ERT), which is reimbursed in Belgium since 2006 [9, 10], and supportive therapies such as (non-)invasive ventilation and physiotherapy. Similar to many other neuromuscular disorders, validated outcome measures are largely lacking in Pompe disease.

Here, we studied the clinical, brain imaging, and genetic features of the Belgian cohort of LOPD patients (*N* = 52), and the sensitivity of different outcome measures, during a longitudinal period of 7 years (2010–2017). One of the outcome measures was the activity limitations measure ActivLim [11–13], which has been evaluated previously in several other neuromuscular disorders, but not specifically in Pompe disease.

## Patients and methods

### LOPD patients

In this retrospective cohort study, we obtained pseudonymized data from LOPD patients from the seven neuromuscular reference centres in Belgium, where all the known LOPD patients are followed (*N* = 52), and from the Pompe disease registry (Sanofi-Genzyme). For patients receiving ERT in Belgium, a regular 6-month standardised follow-up and inclusion in a neuromuscular reference centre is mandatory. This allowed us to collect reliable data. We gathered the following patient data: gender, current age, age at onset, symptoms at onset, current disease stage, gene mutations, age at start of ERT, (non)-invasive ventilation and start-up age, serum creatine kinase (CK) (normal value for males < 190 U/l, females < 174 U/l), brain imaging either computed tomography (CT) including CT-angiography or magnetic resonance imaging (MRI) including MR-angiography. Furthermore, we studied the following functional data in the period between 2010 and 2017: forced vital capacity (FVC) in sitting and supine position (percentage of the predicted normal value), 6-min walk distance (6MWD), 10-m walk test (10MWT), Medical Research Council (MRC) sum score (shoulder abduction, elbow flexion, elbow extension, hip flexion, knee extension and knee flexion on both sides; each muscle group is scored between 0 and 5, maximum total score is 60), and the ActivLim (acronym of “ACTIVity LIMitations”) questionnaire [11–13] . The latter comprises 22 daily activities to be rated by the patients as impossible, difficult or easy. It is a validated measure of daily activity limitations for patients with neuromuscular diseases [14]. A higher ActivLim score corresponds to a better functional level. The ActivLim score was computed using the Rasch model. Genetic analyses comprised both Sanger sequencing and MLPA analysis to also detect deletions and duplications. The genetic results were compared with the Pompe disease *GAA* variant database [8] and current literature [15].

### Statistical analysis

We performed all statistical analyses using SAS software, version 9.4 of the SAS System for Windows. A linear mixed model with correlated random intercept and slope was used to evaluate the evolution of each variable as a function of the years since onset. Restricted cubic splines with four knots [16] were used to allow nonlinearity. Likelihood-ratio tests were used to test the assumption of linearity, and to test if there was evidence for a change over time (in the model assuming linearity, as well as in the model allowing a nonlinear relation). The same methodology was used to verify the relation between the ActivLim score and each of the other variables. Thus, instead of using years since onset, the ActivLim score functioned as predictor in the model. After standardising the variables, the estimate of the slope in the model assuming linearity was reported. This estimate corresponds to a correlation coefficient. Note that this coefficient quantifies the association within a subject. To evaluate the cross-sectional correlation, which answers the question if a patient with on average high ActivLim values has also on average high values for another outcome measure, mean values per subject have been calculated and the correlation based on these mean values was reported.

## Results

### Prevalence of LOPD in Belgium

In Belgium, we identified 52 patients with LOPD, belonging to 48 families. The calculated prevalence of LOPD in Belgium is 3.9 per million, on a current population of 11,431,406 [7].

### Demographic and clinical features of LOPD patients in the Belgian cohort (Table 1)

Twenty-seven patients were female (52%), 25 male (48%). Mean current age was 47.9 years (SD: 15.2 y; *N* = 45), and mean age at symptom onset 28.9 years (range [7 mo – 68 y]; SD: 15.8 y). Two patients had onset of symptoms below 1 year of age (7 and 9 months); however, enzyme activity level and normal cardiac investigations were compatible with LOPD. In 75% of patients (*N* = 39), limb-girdle weakness was the initial symptom of the disease, presenting either as the only symptom or in combination with other abnormalities such as respiratory weakness and/or axial weakness and/or fatigue. One third (*N* = 17) of patients experienced respiratory problems as an initial symptom of the disease, and in 13% (*N* = 7) respiratory symptoms were the only initial symptom. In one patient, the disease presented with fatigue and an increased serum CK-level. Currently, only one of the 45 living patients is wheelchair-dependent, whereas the others are still ambulatory (although some of them use walking aids and/or a wheelchair for longer distances). Brain imaging (either CT of MRI) including imaging of the vessels was performed in 32 patients and revealed abnormalities in eight patients (25%). Small cerebral aneurysm(s) were detected in two patients and a vertebrobasilar dolichoectasia in another two (Table 1).

**Table 1:** Clinical, brain imaging and genetic features of the Belgian cohort of LOPD patients

ID	Gender	Current age (y)	Current disease stage	Age at symptom onset (y)	Symptoms at onset	Duration of ERT (y)	Ventilation (age at startup in y)	Brain imaging	Year of genetic diagnosis (age in years)	***GAA*** mutation 1	***GAA*** mutation 2
1	F	D	D (68y)	42	LW	2	NIV (54y); invasive (68y)	ND	2013 (65y)	c.-32-13 T > G	c.2219_2220del
2	F	66	A	58	R	4	NIV (58y)	Normal (CT)	2014 (60y)	c.-32-13 T > G	c.2331 + 2 T > A
3	F	23	A	14	LW	8	–	ND	2010 (14y)	c.-32-13 T > G	c.1564C > G
4	M	56	A	15	LW, F	13	NIV (39y)	ND	2007 (44y)	c.-32-13 T > G	c.258dupC
5	M	55	A	26	LW, F	2	NIV (53y)	ND	2016 (52y)	c.-32-13 T > G	c.2261dupC
6	F	65	A	13	LW	7	–	ND	2012 (58y)	c.-32-13 T > G	c.258dupC
7	M	44	A	14	LW, F	4	NIV (32y)	ND	2007 (32y)	c.-32-13 T > G	del exon 18
8	M	D	D (age?)	10	R	3	invasive (42y)	ND	2007 (41y)	c.-32-13 T > G	c.956del7
9	F	75	W	49	LW	13	–	Atrophy, mild microvascular white matter lesions (MRI)	2005 (61y)	c.-32-13 T > G	c.525delT
10	M	D	D (age?)	68	R	8	NIV (75y)	ND	NA	c.-32-13 T > G	–
11	M	45	A	11	LW	13	NIV (37y)	Normal (MRI)	2007 (33y)	c.-32-13 T > G	c.1548G > A
12	M	27	A	7	LW	14	–	ND	2002 (20y)	c.-32-13 T > G	c.2331 + 2 T > A
13^a^	F	45	A	36	LW	2	–	ND	NA	c.-32-13 T > G	c.1115A > T
14 ^a^	M	D	D (62y)	NA	R	2	NIV (58y)	ND	2005 (60y)	c.-32-13 T > G	–
15^a^	M	55	A	30	R	13	NIV(36y)	ND	2004 (40y)	c.-32-13 T > G	del exon 18
16^a^	M	59	A	52	NA	11	–	Normal (MRI)	2004 (44y)	c.-32-13 T > G	del exon 18
17	F	64	A	51	R, LW	9	–	Normal (MRI)	2008 (53y)	c.-32-13 T > G	c.258dupC
18	F	27	A	11	NA	12	–	Normal (MRI)	2006 (14y)	c.-32-13 T > G	c.258dupC
19	F	48	A	35	R, LW, AW	10	–	Normal (MRI)	2008 (37y)	c.-32-13 T > G	del exon 18
20	F	50	A	31	R, LW, AW	9	–	Moderate ventriculomegaly (MRI)	2009 (40y)	c.-32-13 T > G	c.692 + 1G > T
21^b^	M	50	A	20	R, LW, AW	10	–	Normal (MRI)	2008 (39y)	c.-32-13 T > G	**c.1610_1611delA**
22^b^	M	47	A	37	R, LW, AW	9	–	Normal (MRI)	2008 (36y)	c.-32-13 T > G	**c.1610_1611delA**
23	M	34	A	15	NA	7	–	Normal (MRI)	2007 (22y)	c.-32-13 T > G	c.2608C > T
24	M	15	A	7 mo	NA	12	–	Aspecific T2 hyperintensity in right thalamus (MRI)	2007 (3y)	c.-32-13 T > G	del exon 18
25	F	41	A	17	LW, R	8	–	Normal (MRI)	2007 (29y)	c.-32-13 T > G	c.1655 T > C
26	M	10	A	2.5	R, LW	6	–	Normal (MRI)	2013 (4y)	C.2608C > T	c.1839G > C
27	F	59	A	43	R, LW, AW	5	–	Normal (MRI)	2013 (53y)	c.-32-13 T > G	c.2608C > T
28	F	35	A	28	R, LW, AW	3	–	Normal (MRI)	2015 (31y)	c.-32-13 T > G	c.1121G > A
29	F	23	A	9 mo	LW	14	–	Normal (MRI)	2000 (4y)	c.-32-13 T > G	c.923A > C
30	M	63	A	39	R	13	NIV (40 y)	Vertebrobasilar dolichoextasia, hypointensities in basal ganglia (SWI) (MRI)	2007 (51y)	c.-32-13 T > G	c.258dupC
31	F	50	A	35	LW	13	–	Normal (MRI)	2007 (38y)	c.-32-13 T > G	c.1548G > A
32	F	73	A	28	LW	13	–	ND	2007 (61y)	c.-32-13 T > G	c.1710C > G and c.1923G > A
33	F	63	A	44	LW	11	–	Normal (CT)	2008 (52y)	c.-32-13 T > G	del exon 18
34^c^	M	68	A	45	LW, ptosis left side	12	NIV (60 y)	Aneurysm right vertebral artery (MRI)	2007 (66y)	c.-32-13 T > G	c.1681_1699dup19
35^c a^	F	D	D (78y)	44	LW, AW	–	NIV (72 y)	Vascular leukoencephalopathy (CT)	2007 (72y)	c.-32-13 T > G	c.1681_1699dup19
36	M	47	A	25	LW	11	–	Multiple small aneurysms (right ACI and left ACA) (MRI)	2008 (36y)	c.-32-13 T > G	c.1075G > A
37^d^	M	45	A	17	LW	10	–	Normal (MRI)	2009 (35y)	c.-32-13 T > G	c.482_483del
38	F	41	A	27	HyperCK, F	9	–	Normal (MRI)	2009 (31y)	c.-32-13 T > G	c.525delT
39^d^	F	49	A	42	LW	7	–	Normal (MRI)	2011 (41y)	c.-32-13 T > G	c.482_483delCC
40	F	35	A	18	LW	3	–	Normal (MRI)	2016 (32y)	c.-32-13 T > G	c.2261dupC
41	M	55	A	42	LW	2	NIV (53 y)	Normal (MRI)	2017 (53y)	c.-32-13 T > G	c.2608C > T
42	M	59	A	51	LW	5	–	ND	2014 (53y)	c.-32-13 T > G	c.258dup
43	F	63	A	25	LW	11	–	ND	2011 (54y)	c.-32-13 T > G	**c.186dup11**
44	F	62	A	44	LW	7	–	ND	2007 (51y)	c.-32-13 T > G	c.1115A > T
45	F	D	D (65y)	44	LW, R	7	NIV (55y)	Normal (CT)	2008 (57y)	c.-32-13 T > G	c.701C > G
46	M	58	A	27	LW	8	NIV (54y)	ND	2012 (51y)	c.-32-13 T > G	c.258dup
47	M	53	A	29	LW	11	NIV (41y)	Normal (CT)	2008 (42y)	c.-32-13 T > G	c.525delT
48	M	46	A	9	LW	14	NIV (38y)	Normal (MRI)	2007 (34y)	c.1-45 T > G	c.2608C > T
49	F	45	A	20	R	2	–	Vertebrobasilar dolichoextasia (MRI)	2017 (43y)	c.-32-13 T > G	c.525delT
50	M	40	A	36	LW	3	NIV (37y)	ND	2017 (38y)	c.-32-13 T > G	c.1115A > T
51 ^a^	F	21	A	18	LW, F	4 mo	–	ND	2019 (21y)	c.-32-13 T > G	c.1548G > A
52	F	D	D (age?)	NA	NA	NA (until D)	NIV (49y)	ND	NA	NA	NA

*A* ambulatory; *ACA* anterior cerebral artery, *ACI* internal carotid artery, *AW* axial weakness, *CT* computed tomography, *D* deceased, *ERT* enzyme replacement therapy, *F* female, *F* fatigue, *GAA* alpha-glucosidase gene, *HyperCK* increased creatine kinase in blood, *LW* limb-girdle weakness, *M* male, *mo* months, *MRI* magnetic resonance imaging, *NA* not available, *ND* not done, *NIV* non-invasive ventilation, *R* respiratory weakness, *SWI* susceptibility weighted imaging, *W* wheelchair-bound, *y* years. ^a^, patients not included in statistical analysis of functional tests (ActivLim data lacking). Letters in superscript indicate corresponding family members. New mutations are indicated in bold

All patients were treated with enzyme replacement therapy (ERT), except one patient because of advanced age and severe clinical stage at the time of ERT introduction (patient 35, Table 1). Mean age at start-up of ERT was 41.2 years (SD: 15.9 y) and mean duration of ERT treatment up to now was 8.1 y (SD: 4.0 y). Nineteen patients (37%) were treated with non-invasive ventilation (NIV) starting at a mean age of 49.5 years (range [32–75], SD: 11.9 y), with a mean duration of 15 years (SD: 10.2 y) after symptom onset. One of these patients subsequently needed invasive ventilation at the age of 68 years, 14 years after starting NIV (patient 1, Table 1). Another patient was treated immediately with invasive ventilation at age 42 years, due to respiratory failure (patient 8, Table 1). Interestingly, this patient presented with respiratory difficulties as a first symptom at age 10 years. Mean serum-CK levels prior to ERT were 686 U/l (range [65–2246], SD: 510 U/l), whereas the current mean serum-CK values (under ERT) were 399 U/l (range [19–1303], SD: 326 U/l).

### Diagnosis and genetic results in LOPD patients in the Belgian cohort (Table 1)

In our cohort, all patients were compound heterozygotes, none of them carried homozygous mutations in *GAA*. The majority (49/51, 96%) harboured the frequent c.-32-13 T > G (IVS1) mutation in the *GAA*-gene. We identified a novel *GAA* mutation in three patients belonging to two different families: c.1610_1611delA (patients 21 and 22) and c.186dup11 (patient 43, Table 1). In two patients, we identified only one mutation in *GAA*, but we could not find a second mutation (patients 10 and 14, Table 1). However, the diagnosis in these patients was proven by reduced GAA enzymatic activity in blood and muscle tissue. Mean age at genetic diagnosis was 40.8 years (SD: 16.4 y), and diagnostic delay between symptom onset and genetic diagnosis 12.9 years (range [0–45], SD: 10.8 y).

### Data on motor performances (6MWD, 10MWT, MRC sum score), respiratory function (FVC) and ActivLim score in LOPD patients

The last measured mean value for 6MWD was 385.1 m (SD: 167.7), for 10MWT 9.2 s (SD: 6.9), for the MRC sum score 49.7 (SD: 6.7), FVC sitting 73.8% (SD: 25.7) and FVC supine 57.3% (SD: 25.3). For the patients using (non-)invasive ventilation (*N* = 19), mean FVC sitting was 51.1% (SD: 24.8) and mean FVC supine 33.0% (SD: 15.5).

**Fig. 1:**
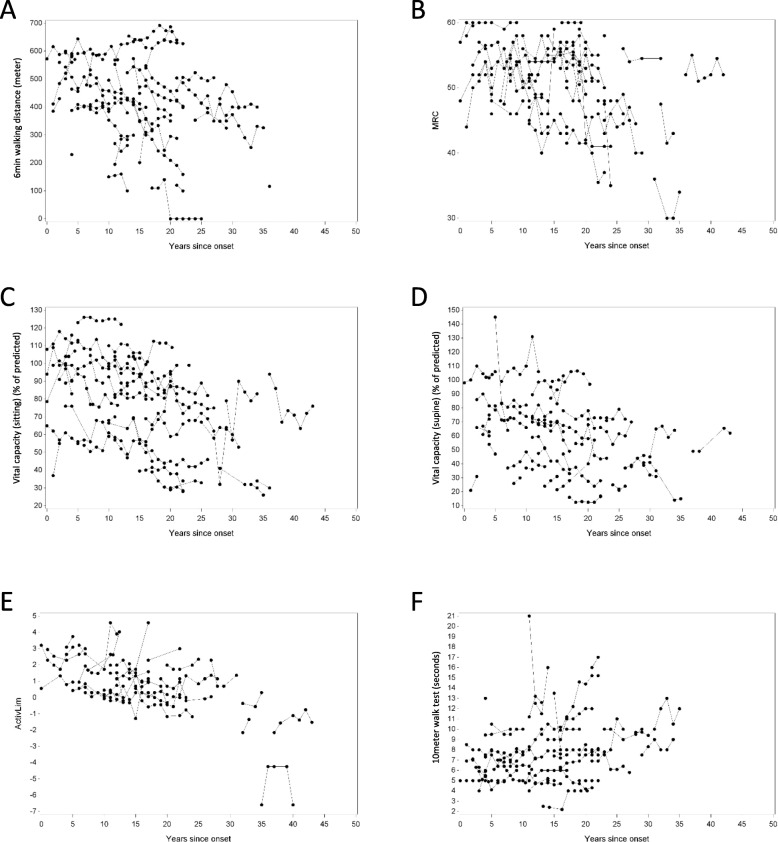
Panels **a** through **f** show the individual data points of the mentioned outcome parameters over time (years since symptom onset). Panel **a** 6MWD (in meters); Panel **b** MRC sum score (maximum score of 60); Panel **c** and **d** FVC (% of predicted outcome); Panel **e** Activlim score; Panel **f** 10MWT (in seconds)

**Fig. 2:**
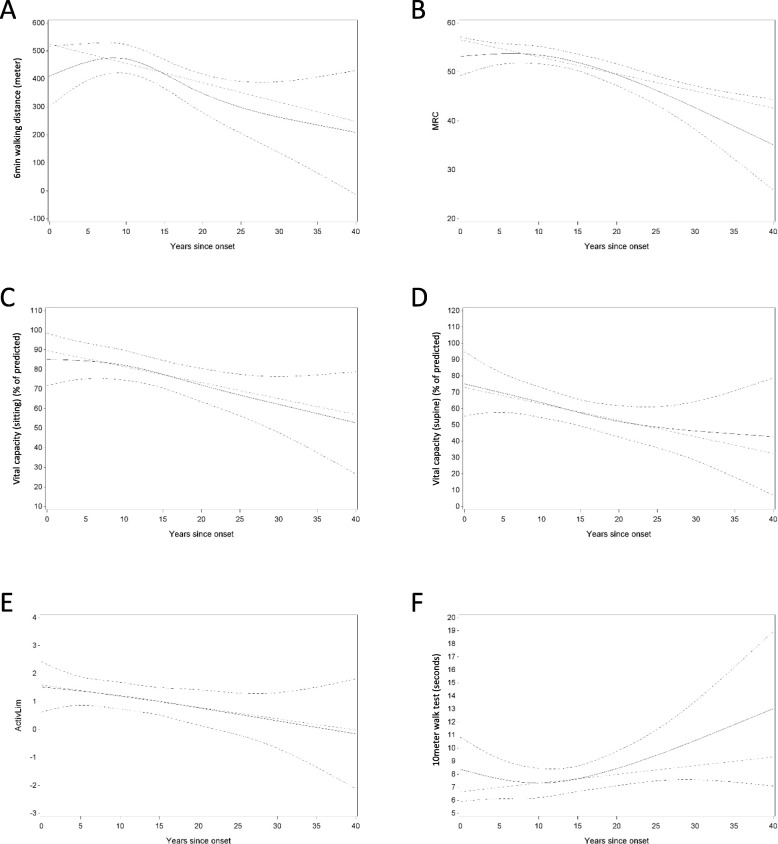
Panels **a** through **f** show the evolution of the mentioned outcome parameters over time (years since symptom onset), with the upper and lower dotted lines indicating the 95% confidence interval. Dotted green line shows the expected linear evolution of each parameter over time. Panel **a** 6MWD (in meters); Panel **b** MRC sum score (maximum score of 60); Panel **c** and **d** FVC (% of predicted outcome); Panel **e** Activlim score; Panel **f** 10MWT (in seconds)

Longitudinal data on 6MWD, 10MWT, MRC sum score, FVC sitting, FVC supine and ActivLim score were available for 43, 41, 44, 48 and 33 out of 51 patients, respectively (Fig. 1). Most observations were made during the first 25 years after disease onset: 195/222 (88%) for 6MWD, 182/210 (87%) for 10MWT, 186/213 (87%) for MRC sum score, and for FVC sitting 219/253 (87%), FVC supine 159/186 (85%) and ActivLim score 126/151 (83%). For some of the outcome measures there seems to be an improvement initially, before deterioration becomes obvious. We calculated the evolution over time (years after symptom onset) for the different outcome measures (6MWD, 10MWT, MRC sum score, FVC sitting and supine, ActivLim score). For the 6MWD, MRC sum score, FVC sitting and FVC supine, we observed a significant decrease over time (*p* = 0.0002, *p* = 0.0001, *p* = 0.0077, *p* = 0.0151, respectively) (Fig. 2, Table 2). For the ActivLim score, we observed a non-significant linear decrease over time (*p* = 0.0938). Excluding one outlier, there was also no significant change for the 10MWT over time (*p* > 0.05) (Fig. 2, Table 2).

**Table 2:** Significance levels of outcome measures over time

	nonlinearity	Evolution over time	
		linear	nonlinear
ActivLim	0.9792	0.0938	0.2405
6MWD	0.0062	0.0102	0.0002
10MWT	0.1195	0.2676	0.0646
MRC sum	0.0431	0.0006	0.0001
FVC sitting	0.5594	0.0077	0.0160
FVC supine	0.7773	0.0151	0.0406

*Results (p-values) likelihood-ratio tests from mixed models evaluating the evolution of each of the outcome measures as a function of years since onset. Nonlinearity: is there evidence that the relation is nonlinear (nonlinearity). Linear, nonlinear: is there evidence that the measure changes over time (assuming linearity, and allowing nonlinearity, respectively).*

### Correlation of longitudinal motor and respiratory outcome measures (6MWD, 10MWT, MRC sum score, FVC sitting, FVC supine) with the ActivLim score

Two correlations were performed: a cross-sectional correlation based on the mean values per subject (differences in number of measurements were ignored as simplification) and the within-subject correlation as derived from the mixed model. For the 6MWD, there is a significant correlation for the mean values per subject and the within-subject measurements (*p* = 0.0005, *p* = 0.0018, respectively). For the 10MWT, we also found significant correlations for these two measures (*p* = 0.0010, *p* = 0.0001, respectively), after exclusion of one outlier. For MRC sum score there was a significant correlation when comparing means per subject (*p* = 0.0240), but not within-subject (*p* > 0.05). For FVC values, both sitting and supine, there was a significant correlation when comparing means per subject (*p* = 0.0082, *p* = 0.0044, respectively), but within-subject only for supine FVC (*p* = 0.0346).

## Discussion

### LOPD is a rare disease and associated with a long diagnostic delay

We established a prevalence of LOPD in Belgium of 3.9 per million or 1:256,000. An overall estimated prevalence of 1:283,000 has been reported in Europe [17, 18]. A cohort study in Austria revealed an even lower prevalence of 1:351,000 of Pompe disease (including both LOPD and infantile forms) [18]. Thus, LOPD is a rare disease, and the low prevalence may be one explanation for the long diagnostic delay of almost 13 years in our study, 7.4 years in the Austrian cohort [18], about 5 years in a Chinese patient group [19] and 3.3 years in an Iranian cohort [20]. Furthermore, the clinical presentation is variable and can be mild and non-specific, especially at the beginning of the disease. Some patients are pauci- or asymptomatic and only present with hyperCKemia or fatigue. Importantly, in 13% of our patients, respiratory symptoms were the only initial symptom of the disease. Early recognition and diagnosis of Pompe disease is important since treatment (ERT) is available and it has been shown that ERT is more efficient in early disease stages [9]. In later disease stages, patients with LOPD mainly show limb-girdle and axial muscle weakness with or without respiratory symptoms, which was also the case in our study population. In order to decrease the long diagnostic delay in LOPD patients, awareness on LOPD as a rare disease should be significantly raised further.

### Cerebral (vascular) abnormalities in LOPD

Brain imaging (either CT or MRI) including imaging of the vessels revealed abnormalities in 25% (*N* = 8) of the patients. Most anomalies were nonspecific findings but in two patients small cerebral aneurysm(s) (2/32; 6.5%) were detected and a vertebrobasilar dolichoectasia in another two (2/32; 6.5%). Recent studies in LOPD showed a relative higher prevalence of intracranial abnormalities than we found in our study. Montagnese et al. [21] revealed the presence of aneurysms in 9.5% of LOPD patients (2/21) and a vertebrobasilar dolichoectasia in 47% (10/21). Musumeci et al. [22] also reported a higher prevalence of intracranial aneurysms (3/21; 14%) and a vertebrobasilar dolichoectasia (11/21; 52%) in LOPD patients. Furthermore, in both studies a relatively high frequency of lacunar encephalopathy was reported (62 and 57%, respectively). Management guidelines for aneurysms and other intracranial abnormalities in Pompe disease are lacking; mostly, current general recommendations for surgery or for radiological follow-up are used. As intracranial vascular anomalies can lead to serious complications, performing brain imaging (preferably MRI with MR angiography) in LOPD patients, at least once at the time of diagnosis, is recommended [23]. If aneurysms are detected and warrant no treatment, brain imaging should be repeated every 6–12 months, as indicated by the guidelines of the American Stroke Association for brain aneurysms in general [24].

### The 6MWD, MRC sum score, FVC sitting and FVC supine are sensitive longitudinal outcome measures in LOPD, in contrast to the ActivLim score and 10MWT

Defining appropriate outcome measures is important to understand the disease course and to better design current and future clinical trials in LOPD. We obtained longitudinal data on distinct outcome measures in our study population between 2010 and 2017, irrespective of age, clinical status, disease duration or therapy duration. We studied both motor and respiratory parameters. FVC is measured both in sitting and supine position, because diaphragmatic involvement, which frequently occurs in LOPD, can be measured by a significant difference of more than 25% between the two FVC values. For the 6MWD, MRC sum score, FVC sitting and FVC supine, we measured a significant decrease over time in LOPD patients, even though they were treated with ERT. This deterioration was not revealed by the 10MWT and ActivLim score, although our data suggested a trend towards a decline.

Vandervelde et al. [12] showed that in several other neuromuscular diseases, such as Duchenne muscular dystrophy, other muscular dystrophies and Charcot-Marie-Tooth neuropathy, the ActivLim score was possibly even more sensitive than patients’ self-perception to report change in functional status (overall better, stable or worse). In Batcho et al. [13], the ActivLim score (analysed with the rating scale Rasch model) was validated in a more heterogeneous group of neuromuscular disorders in the longitudinal monitoring of functional status. However, these two studies did not include patients with LOPD. Van der Beek et al. [25] developed a Pompe-specific scale, i.e. the Rasch-built Pompe-specific activity (R-PAct) scale. Difficulty of tasks incorporated in different questionnaires differs substantially between neuromuscular diseases; this could be one reason why we do not find a significant change in the ActivLim score in LOPD. In Lachmann and Schoser [26], the minimally clinical important difference deterioration in FVC in non-treated Pompe patients would possibly be felt after 2 years, for the 6MWD this would be 9 years. This might also be an explanation why there is a change in some outcome measures, but not in the ActivLim score.

Not all changes in outcome measurements in neuromuscular diseases result in a change in functionality [26], the relation between motor function and activity level is not necessarily straightforward [27, 28]. Declines in walking distance, speed or forced vital capacity are not necessarily clinically significant or even perceived as a decline by the patient. Therefore, activity limitations should be assessed separately in patients with neuromuscular diseases, but in LOPD patients the ActivLim score may not be the best patient reported outcome measure to asses this.

When comparing mean values of respiratory function and 6MWD, findings in our LOPD cohort are comparable to earlier described LOPD patient groups [10, 18, 29, 30]. In the Austrian cohort, 6MWD during ERT was 373.5 m [18], and Kuperus et al. [30] showed that the median 6MWD changed from 376 to 416 m over a period of 5 years following ERT onset. They measured an increase in 6MWD during the first year after treatment onset, and a decline from then on. In the meta-analysis of Schoser et al. [10], where distances between 246 and 660 m were measured, the largest improvement of the 6MWD was measured the first 20 months after start of ERT. Similarly, in our cohort, the 6MWD declined over the years, but we collected data irrespective of ERT and of ERT duration.

The 6-min walk distance (6MWD) is most commonly used to measure exercise capacity in LOPD [26, 31]. This capacity may be limited by multiple causes, such as peripheral muscle weakness and respiratory function. Furthermore, this may vary between patients. In Duchenne and Becker muscular dystrophy, as well as in some pulmonary diseases, the 6MWD is a validated outcome measure, often used in combination with other tests, such as quantitative muscle MRI and Motor Function Measure [26]. Similarly to previous longitudinal cohorts in neuromuscular diseases [32], we showed that the 6MWD was a good outcome parameter in LOPD.

### Limitations and strengths of the study

Because of the retrospective nature of the study, there were some missing data for different outcome measures, the patient population was heterogeneous concerning current age and disease duration since data were collected from a fixed timeframe (2010–2017), and there was no comparison with an untreated control group since most data in the study were obtained from treated LOPD patients.

Despite these shortcomings, we performed a detailed clinical, imaging and genetic characterisation of all Belgian LOPD patients and identified useful longitudinal outcome measures in LOPD that can be applied in future clinical trials.

## Conclusions

Awareness on LOPD should even be further increased, as we found a long diagnostic delay as described in other populations. As ERT is more effective in the beginning of the disease, early start of therapy is important. In the follow-up of patients, physical outcome measures and patient reported outcome measures are needed, as the relation between these two is not straightforward. The 6MWD, but not the ActivLim score, is a sensitive outcome measure to follow up LOPD patients.

## Abbreviations

10MWT: 10 m walk test
6MWD: 6 min walking distance
CK: Serum creatine kinase
ERT: Enzyme replacement therapy
FVC: Forced vital capacity
GAA: Acid alpha-glucosidase
GSD2: Glycogen storage disease type 2
IOPD: Infantile onset Pompe disease
LOPD: Late onset Pompe disease
MRC: Medical Research Council
NIV: Non-invasive ventilation
